# Drinking Water Management: Health Risk Perceptions and Choices in First Nations and Non-First Nations Communities in Canada

**DOI:** 10.3390/ijerph110605889

**Published:** 2014-05-30

**Authors:** Diane Dupont, Cheryl Waldner, Lalita Bharadwaj, Ryan Plummer, Blair Carter, Kate Cave, Rebecca Zagozewski

**Affiliations:** 1 Department of Economics, Environmental Sustainability Research Centre, Brock University, St. Catharines, ON L2S 3A1, Canada; 2 School of Public Health, University of Saskatchewan, Saskatoon, SK S7N 5E5, Canada; E-Mails: cheryl.waldner@usask.ca (C.W.); lalita.bharadwaj@usask.ca (L.B.); 3 Environmental Sustainability Research Centre, Brock University, St. Catharines, ON L2S 3A1, Canada; E-Mail: rplummer@brocku.ca; 4 Stockholm Resilience Centre, University of Stockholm, Stockholm, SE-106 91, Sweden; E-Mail: rplummer@brocku.ca; 5 Department of Environment and Resource Studies, University of Waterloo, Waterloo, ON N2L 3G1, Canada; E-Mail: blcarter@uwaterloo.ca; 6 Institute for Water, Environment, and Health, United Nations University, Hamilton, ON L8P OA1, Canada; E-Mail: kate.cave@unu.edu; 7 Safe Water for Health Research Team, University of Saskatchewan, Saskatoon, SK S7N 5E5; Canada; E-Mail: mailto:r.zagozewski@usask.ca

**Keywords:** bottled water expenditures, health risk beliefs, First Nations, generalized estimating equations, odds-ratios

## Abstract

The relationship between tap water and health has been a topic of public concern and calls for better management in Canada since well-publicized contamination events in two provinces (Ontario and Saskatchewan) in 2000–2001. This study reports the perspectives on health risks from tap water and corresponding use of, and spending on, bottled water in a number of different communities in Canada. In 2009–2010, four First Nations communities (three from Ontario and one from Saskatchewan) and a geographically diverse sample of non-First Nations Canadians were surveyed about their beliefs concerning health risks from tap water and their spending practices for bottled water as a substitute. Responses to five identical questions were examined, revealing that survey respondents from Ontario First Nations communities were more likely than non-First Nations Canadians to believe bottled water is safer than tap water (OR 1.6); more likely to report someone became ill from tap water (OR 3.6); more likely to express water and health concerns related to tap water consumption (OR 2.4); and more likely to spend more on bottled water (OR 4.9). On the other hand, participants from one Saskatchewan First Nations community were less likely than non-First Nations Canadians to believe that someone had become ill from drinking tap water (OR 3.8), less likely to believe bottled water is safer than tap (OR 2.0), and less likely to have health concerns with tap water (OR 1.5). These differences, however, did not translate into differences in the likelihood of high bottled water expenditures or being a 100% bottled water consumer. The paper discusses how the differences observed may be related to water supply and regulation, trust, perceived control, cultural background, location, and past experience.

## 1. Introduction

The relationship between tap water and health has been a topic of public concern whereby Canadian citizens have called for better management. Contamination events in Walkerton, Ontario and North Battleford, Saskatchewan that occurred more than ten years ago led to many illnesses and, in the case of Walkerton, seven deaths [1]. Nonetheless, tap water problems have continued to plague most parts of Canada. For example, 1766 boil-water advisories were in effect in 2008 [2]. More recently, in 2012 and 2013, respectively, 40,000 citizens in Prince Albert, Saskatchewan and 1.3 million residents in Montreal, Quebec, were advised to boil their water [3]. The effect on First Nations communities is of particular concern. In 2005, residents of Kashechewan, a Northern Ontario First Nations community, were evacuated because of the presence of high levels of *E. coli* in their drinking water [4]. On January 31, 2013 Health Canada reported that 113 First Nations communities were on boil water advisories [5]. Six years earlier Health Canada had reported on 117 advisories—79% of which were in effect for a year and 25% for more than a year [6]. Despite efforts by the Government of Canada, provincial governments, and First Nations communities, Harden and Levalliant [7] note, “the fact remains that unsatisfactory access to safe drinking water persists for many First Nations people…”.

A recent national assessment to evaluate water and wastewater systems on First Nations’ reserves for Aboriginal Affairs and Northern Development Canada (AANDC) provided a snapshot of the state of these systems [8]. In viewing all Canadian First Nations reserves together, 39% of water systems were classified as high risk, 34% as medium risk, and 27% as low overall risk. In this context, the report noted that systems identified as high risk have “deficiencies (that)… may lead to potential health and safety or environmental concerns” while systems identified as low risk operate with “minor deficiencies” [8]. For the province of Saskatchewan, 103 First Nations water systems were inspected and 26% were classified as high risk and 28% as low risk respectively. Communities that obtained water from Municipal Type Agreement (MTA) systems—generally, these involve the piping of water to a First Nations reserve from an adjacent municipal source—were found to have the lowest risk, followed by groundwater systems in which drinking water is derived from a groundwater source. In Ontario First Nations, 158 water systems were inspected and 46% were categorized as high risk and 16% as low overall risk. These low-risk systems included a mix of groundwater and surface water systems with some Municipal Type Agreement systems. Since individual provinces are responsible for their natural resources, which includes drinking water, a number of provinces have created and employed drinking water quality assessment tools that are tailored to their particular locale; however, as a result, there is no comparable national-level data available for non-First Nations communities across Canada [9].

This study had two key objectives. The first was to investigate whether there are differences in beliefs about health risks from tap water and bottled water purchases of residents in four First Nations communities (three in Ontario and one in Saskatchewan) and a geographically diverse sample of non-First Nations Canadians across Canada. The second objective was to determine whether observed differences in health concerns translate into health risk avoidance expenditures and responses. Our ability to survey a diverse set of respondents with identical questions was made possible by networking between researchers across Canada who were individually working in their own regions on issues relating to the topic of water management, health risk perceptions, and consumer responses.

## 2. Material and Methods

### 2.1. Background

Three surveys were conducted that contained a subset of identical questions. The analysis is presented in this paper. The survey methodology employed for each survey is different and is described below.

#### 2.1.1. Survey A

Three Ontario First Nations communities and a consortium of academic and NGO partners joined in undertaking a research project entitled: *First Nations and Source Waters: Understanding Vulnerabilities and Building Capacity for Environmental Governance*. The Six Nations reserve is the most populous First Nations in Canada with a registered population of 24,000 people (13,000 living on reserve). New Credit has a total population of 1788, with 847 on reserve, and is located near Hagersville, ON. The New Credit reserve is located adjacent to the Six Nations reserve. The Oneida First Nations reserve is located along the Thames River, and has a population of 5000 people with 2000 on reserve. The project team collaboratively designed a questionnaire to capture information and perceptions on several aspects of drinking water. A venue-based method for survey distribution to community members was selected and was based on recommendations by First Nations community partners. Academic and community researchers worked together to administer the questionnaire at community events during the summer/fall of 2009 (e.g., fall fair, community picnic, water appreciation day). Respondents were verbally invited to participate in the study and informed consent procedures were explained. A cash incentive was provided for completion of the questionnaire to acknowledge the value of the respondent’s time. The individuals administering the questionnaire worked through each of the items with the participants to ensure accuracy of interpretation and to answer any questions. Permission to conduct the survey was granted by each First Nations community and the Brock University Research Ethics Board (REB #09-262).

#### 2.1.2. Survey B

A community-based participatory research project was initiated to jointly gather information on the nature, availability, utilization, perceptions, and attitudes of community drinking water sources in Muskoday First Nations, Saskatchewan, located 19 km southeast of Prince Albert, SK. It has a registered population of 1583 people with 565 members living on reserve. The project was based on respectful collaboration and principles outlined in [10] which include the application of participative strategies. Over the period January to May 2010, the project team developed and finalized an in-person household survey tool, planned methodology, hired a community research assistant, and recruited participants. The project was initiated in May of the same year, following the training of the community research assistant, whose primary role was to conduct the surveys. The survey was paper based, completed in-person with a household member 18 years or older and was administered from June through to August 2010 by the community research assistant. The Muskoday Lands and Resource officer provided a housing list (208 households) to the research assistant and a convenience sample of 86 households were surveyed representing 41% of community households. Ethical approval was obtained through appropriate community approval processes and the University of Saskatchewan Research Ethics Board (REB #08-197).

#### 2.1.3. Survey C:

A series of focus groups were convened in fall 2007 and winter 2008 to assist with drafting a survey that was administered to a diverse cross-Canada sample of Canadians in order to obtain information about water consumption choices and their relation to health concerns and risk perceptions. The research team followed best practices for the undertaking of surveys [11] and permission was given to conduct the survey by Brock University’s Research Ethics Board (REB #07-320). After a pilot test, the full survey was made available on a private website hosted by Ipsos-Reid during the months of February and March 2009. Respondents were randomly recruited via email from a panel of approximately 125,000 internet-enabled Canadians maintained by Ipsos-Reid. The goal of the researchers was to obtain a minimum of 1000 responses from respondents in such a way that the characteristics of the sample would be comparable to those of the general Canadian population regarding age, income, geographic region, and gender. Ipsos-Reid sent out 5,556 email invitations with links to the survey; recipients were not told the purpose of the survey until they had clicked the link and then asked to provide informed consent through continuation onto the survey website. From the original invitations, 1304 individuals completed the survey, a response rate of 23.5%. Of the remainder, 608 began the survey but did not complete it, and 50 participants activated the survey link after the quota for their geographic region had already been filled.

#### 2.1.4. Summary of Water Sources for Participating Communities

At the time of the survey, Six Nations and Oneida operated their own on-reserve community drinking water systems. Source waters were drawn from the Grand River and an aquifer located below the Thames River for Six Nations and Oneida respectively. Of the 2,674 households in Six Nations, 460 were connected to the community water system. Most (459/460) of the households in Oneida were connected to the community water system. The Muskoday and New Credit First Nations communities had Municipal Type Agreements and received pre-treated drinking water, distributed by pipe from an off-reserve community supply. At the time of the survey, there were 208 housing units on the Muskoday First Nations and 197 of these housing units received piped delivery of treated drinking water from Prince Albert, SK, a nearby city, which draws its drinking water from the North Saskatchewan River. New Credit First Nations community members were served by water drawn from Lake Erie, treated and distributed by pipe from Nanticoke, Ontario.

The majority of non-First Nations Canadians obtained their water from municipal water systems. Each province defines municipal water supplies differently; however, in general, they are centralized water works that serve a prescribed number of individuals within a defined geographic area. According to a Statistics Canada report, 95% of households in Canada’s census metropolitan areas (CMAs) are connected to municipal water supplies [12].

### 2.2. Analysis of Survey Questions

Five identical questions in each survey were asked to elicit the following: beliefs about whether tap water is associated with illness or health concerns, the relative safety of tap and bottled water, the preference to only drink bottled water, and the amount of money spent on bottled water. The five questions, utilized in the surveys, were based on previous studies looking at water consumption choices [13,14,15,16,17]. Specifically, participants were asked to compare bottled and tap water by selecting one of five categories: bottled water is much more safe than tap water, a little safer, about as safe as, a little less safe, and much less safe. Because of the relatively small number of responses from some individual communities, we collapsed this variable to produce a binary outcome: “Bottled is safer” (coded as 1 if respondents answered either that bottled is much more safe than tap water or a little safer than tap water. All other responses were coded as 0). Three binary outcome questions were asked: does a respondent believe that someone in the household has become sick from drinking tap water (“Someone was sick”); the primary reason for choosing to consume bottled water is due to health concerns about tap water (“Health concerns with tap water”); and the respondent drinks 100% bottled water (“100% bottled water drinker”). A final question asked respondents to select one of four categories that best represented monthly bottled water expenditures: less than $50, between $50–$100, between $100–$150, and greater than $150. This was collapsed to produce a binary outcome if the respondent spent $50 or more per month (“High Monthly Expenditures”).

A commercial software program was used for all statistical analyses (IBM SPSS Statistics, version 20). The Kruskall-Wallis test was used to compare the age of participants among the five community/survey groups and between First Nations and non-First Nations participants (Table 1). We then used generalized estimating equations (GEE) with a logit link function and binomial distribution to examine responses of each of the five binary outcomes: “Someone was sick”, “Health concerns with tap water”, “Bottled is safer”, “High monthly expenditures”, and “100% bottled water drinker”. The analysis accounted for potential clustering of responses by geographic region (Western provinces, Ontario, Quebec, Eastern provinces). We used a stepwise forwards approach for the three main effects: Community Surveyed (Three Ontario First Nations, One Saskatchewan First Nations, and non-First Nations Canadians), Age (three categories), and Gender. Variables were retained in the model if the overall Wald test for the variable was significant (*p* < 0.05) or if the variable acted as an important confounder. Finally, we evaluated all possible two-way interactions for all main effects remaining after stepwise model construction and retained any interaction terms where *p* < 0.05. The association between each main effect and each dichotomous outcome in the final models are reported as adjusted odds ratios (OR) and the 95% confidence intervals (95% CI) in Table 2. GEE was also used to compare the frequency of female respondents across the communities surveyed and between First Nations (FN) and non-FN Canadians.

**Table 1:** Summary of respondent age, gender, and responses to questions (% of respective samples (n) *) for each of the 5 participating communities from Ontario First Nations, Saskatchewan First Nations, and an internet-based cross Canada survey.

Variable Name	Survey A (Ontario First Nations)			Survey B (Saskatchewan First Nations)	Survey A Plus Survey B	Survey C (Cross-Canada)
	Six Nations	Oneida	New Credit	Muskoday	First Nations Combined	Non-First Nations
**Gender**						
Male	24.0 (24)	38.0 (28)	41.6 (42)	22.1 (19)	29.3 (113)	50.4 (659)
Female	76.0 (76)	62.0 (62)	58.4 (58)	76.7 (66)	70.7 (283)	49.6 (646)
Total Responses	100	100	101	85	386	1,305
**Age**						
18–24	7.1 (7)	17.0 (17)	10.3 (10)	20.5 (17)	13.5 (51)	8.7 (114)
24–54	63.6 (63)	57.0 (57)	57.7 (56)	59.0 (49)	59.4 (225)	59.5 (774)
≥55	29.3 (29)	26.0 (26)	32.0 (31)	20.5 (17)	27.1 (103)	31.8 (415)
Total Responses	99	100	97	83	379	1,303
**Believe Someone was Sick**						
No	92.0 (81)	78.0 (71)	91.7 (88)	96.5 (83)	89.5 (323)	95.2 (1164)
Yes	8.0 (7)	22.0 (20)	8.3 (8)	3.5 (3)	10.4 (38)	4.8 (59)
Total Responses	88	91	96	86	361	1,223
**Health Concerns With Tap Water**						
No	42.9 (36)	46.8 (37)	79.2 (57)	81.9 (59)	61.6 (189)	74.6 (708)
Yes	57.1 (48)	53.2 (42)	20.8 (15)	18.1 (13)	38.4 (118)	25.4 (241)
Total Responses	84	79	72	72	307	949
**Believe Bottled Water is Safer than Tap**						
No	24.2 (23)	40.7 (33)	65.9 (54)	67.7 (42)	46.5 (152)	57.4 (691)
Yes	75.8 (72)	59.3 (48)	34.1 (28)	32.3 (20)	52.5 (168)	42.6 (512)
Total Responses	95	81	82	62	320	1,203
**High Monthly Bottled Water Expenditures**						
No	69.1 (65)	64.9 (63)	84.1 (69)	93.2 (69)	76.7 (266)	93.2 (841)
Yes	30.9 (29)	35.1 (34)	15.9 (13)	6.8 (5)	23.3 (81)	6.8 (61)
Total Responses	94	97	82	74	347	902
**100% Bottled Water Drinker**						
No	31.0 (31)	54.0 (54)	76.2 (77)	91.9 (79)	62.3 (241)	92.0 (1202)
Yes	69.0 (69)	46.0 (46)	23.8 (24)	8.1 (7)	37.7 (146)	8.0 (105)
Total Responses	100	100	101	86	387	1,307

Note: * The first number presented in each cell is the percentage of responses in a given sample and the second number presented in parenthesis is the number of responses in a given sample.

**Table 2:** Summary of the final multivariable models examining the association between community, age, gender and reported health risk beliefs and choices for bottle water consumption in three groups of Canadian communities reported as odds ratios (OR) adjusted for all other variables in the final models and 95% confidence intervals (95% CI).

Variables Considered in the Final Multivariable Models	Outcomes of Interest				
	Believe Someone was Sick	Health Concerns with Tap Water	Believe Bottled Water is Safer than Tap	High Monthly Bottled Water Expenditures	100% Bottled Water Drinker
**Communities Surveyed**					
Ontario FN *vs.* Non-FN	3.64	2.36	1.63	4.93	9.14
	(2.51–5.28)	(2.01–2.77)	(1.38–1.93)	(3.56–6.83)	(6.91–12.12)
	*p* < 0.001	*p* < 0.001	*p* < 0.001	*p* < 0.001	*p* < 0.001
Non-FN *vs.* Saskatchewan FN	3.84	1.49	2.00	1.14	0.81
	(3.45–4.17)	(1.28–1.75)	(1.82–2.22)	(0.77–1.67)	(0.63–1.05)
	*p* < 0.001	*p* < 0.001	*p* < 0.001	*p* = 0.53	*p* = 0.11
Ontario FN *vs.* Saskatchewan FN	13.9	3.55	3.29	5.59	7.41
	(9.26–20.8)	(3.54–3.56)	(3.04–3.56)	(5.16–6.02)	(7.19–7.63)
	*p* < 0.001	*p* < 0.001	*p* < 0.001	*p* < 0.001	*p* < 0.001
**Age**					
18–24 *vs.* ≥55	4.60		4.11	2.83	0.68
	(3.76–5.62)	na	(2.96–5.71)	(1.92–4.19)	(0.54–0.85)
	*p* < 0.001		*p* < 0.001	*p* < 0.001	*p* < 0.001
25–54 *vs.* ≥55	2.19		2.08	1.48	0.94
	(1.95–2.46)	na	(1.90–2.27)	(1.31–1.66)	(0.71–1.23)
	*p* < 0.001		*p* < 0.001	*p* < 0.001	*p* = 0.65
18–24 *vs.* 25–54	2.10		1.98	1.92	0.71
	(1.64–2.67)	na	(1.37–2.86)	(1.39–2.65)	(0.60–0.83)
	*p* < 0.001		*p* < 0.001	*p* < 0.001	*p* < 0.001
**Gender**					
Female *vs.* Male	1.51			1.26	
	(1.04–2.19)	na	na	(1.08–1.47)	na
	*p* = 0.031			*p* = 0.003	

## 3. Results and Discussion

### 3.1. Descriptive Statistics

Table 1 shows descriptive statistics for responses to the five questions, as well as for age and gender of respondents. Reported age differed across the five participating communities/surveys (*p* = 0.007) and between First Nations and non-First Nations respondents (*p* = 0.001). However, most respondents were within the age range of 25–54 in each of the three surveys. Survey respondents from First Nations communities were more likely to be female (high of 78% in Muskoday and low of 58% in New Credit) as compared to a more even gender distribution for the respondents of the cross-Canada survey (Survey C) (*p* < 0.0001).

A relatively small proportion of respondents from the three surveys believed that someone became ill following the consumption of household tap water (Table 1). However, 22% of respondents from Oneida First Nations reported that someone had gotten sick from drinking household tap water.

Health concerns about tap water, which lead to the use of bottled water were common in the Six Nations (57.1%) and Oneida (53.2%) communities. A smaller percentage of respondents from the Muskoday (18.1%) and New Credit (20.8%) First Nations, as well as the cross-Canada survey (25.4%), reported health concerns about tap water, which lead to bottled water consumption.

A large number (75.8%) of survey respondents from Six Nations believed bottled water is safer than tap water. Smaller proportions of survey respondents from the Oneida (59.3%), New Credit (34.1%), and Muskoday First Nations (32.3%), and the cross-Canada survey (42.6%) reported that bottled was safer to drink.

Six Nations also had the highest percentage of participants that reported drinking 100% bottled water (69%), but these participants did not report high monthly expenditures. Exclusive bottled water consumption was reported by 8% of Muskoday and cross Canada survey respondents.

High monthly expenditures (>$50 per month) were reported in 35.1% of respondents from Oneida; however, the majority of respondents from all surveys did not report high monthly expenditures. A small percentage (6%) of survey respondents from Muskoday and across Canada reported high monthly expenditures.

### 3.2. Differences across Communities in Water Quality Perception, Health, and Bottled Water Use

After accounting for potential similarities within geographic region and the age and gender of respondents in the final multivariable model, there were significant differences among the communities surveyed (Ontario First Nations, Saskatchewan First Nations and Non-First Nations Canadians) for each of the outcomes of interest in this study (Table 2). Ontario First Nations communities were more likely to believe someone had gotten sick from drinking tap water (OR 3.6), more likely to have health concerns with drinking tap water (OR 2.4), and more likely to believe bottled water was safer than tap water when compared to cross-Canada non-First Nations survey respondents (OR 1.6). These differences in perceptions appeared to carry over to actions since Ontario First Nations respondents were more likely to spend >$50 per month on bottled water (OR 4.9) and more likely to consume no tap water (be a 100% bottled water drinker) (OR 9.1) than the survey respondents of the cross-Canada survey.

In contrast after accounting for other risk factors in the final models, respondents in the Saskatchewan First Nations community were less likely to report someone had become sick from drinking tap water (OR 3.8), less likely to report health concerns with tap water (OR 1.5), and less likely to believe bottled water was safer than tap when compared to the cross-Canada non-First Nations survey respondents (OR 2.0). However, in contrast to the findings described above for Ontario First Nations communities and non-First Nations respondents, the differences in perceptions between Saskatchewan First Nations and non-First Nations respondents did not translate into differences in either consumption choices (being a 100% bottled water user) or reported expenditures for bottled water.

While age was not a significant factor in explaining differences in health concerns with tap water (*p* = 0.41), it was important for the other four outcomes (Table 2). Respondents between the ages of 18 and 24 were more likely (OR 4.6) than those over 55 years of age to believe that someone had become sick from drinking tap water and also more likely (OR 4.1) to believe that bottled water was safer than tap water. They were also more likely (OR 2.8) to be in the high monthly bottled water expenditures category, but less likely (OR 1.5) to be a 100% bottled water drinker.

Gender was an important factor for two of the five outcomes (Table 2). Women were more likely (OR 1.5) to believe that someone had gotten sick from drinking tap water and they were more likely (OR 1.3) to report high monthly bottled water expenditures. Gender was not associated with health concerns with tap water (*p* = 0.48), reporting that bottled water is safer than tap water (*p* = 0.41), and being a 100% bottled water drinker (*p* = 0.19).

### 3.3. Discussion

Since the water contamination events in Ontario and Saskatchewan that occurred a decade ago, recognition of the fragmentation of water governance in Canada has become a focus for researchers interested in the safety of water supplies for communities across Canada [18,19,20]. Previous work has used primary data to examine water quality and health risk perceptions for non-First Nations Canadians [17] while secondary data from the Canadian 2001 Aboriginal Peoples Survey has allowed researchers to look at First-Nations communities [19]. The current research is the first to use primary data collected from identical survey questions aimed specifically at the goal of comparing perceptions of water quality from both First Nations and non-First Nations communities in Canada.

Ontario First Nations communities were more likely than the participants from the cross-Canada survey to believe someone had gotten sick from drinking tap water. They were also more likely to spend large amounts on bottled water and to report drinking 100% bottled water. In contrast, participants from the Saskatchewan First Nations community were less likely to believe that someone had become ill from drinking tap water, to believe bottled is safer, or to have health concerns with tap water than those from the cross-Canada survey. However, these beliefs did not translate into differences in the likelihood of reporting drinking 100% bottled water or having high monthly bottled water expenditures. Factors such as trust in institutions, service satisfaction and loss of control could potentially account for these findings.

First, trust in institutions is an important determinant of risk perception and has been shown to influence the acceptability of potential hazards and the acceptability of regulators’ decisions [21,22]. Imposed government regulations and the perennial issue of high risk water supplies in First Nations communities might be contributing factors to the disparity observed in survey participants’ perspectives among First Nations communities and between some First Nations and non-First Nations communities. First Nations and non-First Nations communities fall under different jurisdictions with respect to water supply and management; federal in the first case and provincial or municipal in the second. In the last few years, provincial governments in Alberta, Quebec, Manitoba and Ontario have introduced revised legislation with or without water quality standards and monitoring [20]. In June 2013 the Federal government’s Safe Drinking Water For First Nations Act (2012) passed into law to allow for the development of federal regulations regarding the protection of drinking water sources on First Nations’ lands [23].

Second, differences in service satisfaction and the inequity of water supply service among First Nations and non-First Nations could also play a role in different perceptions noted in this study [16,17]. Walters *et al.* [20] recently compared risks related to source water, system design, operating, reporting, and operator expertise in different Ontario drinking water systems. They found that … “none of the non-First Nations communities scored above low risk” [20] and that there are “…significant differences in service standards among First Nations and non-First Nations communities” [20]. They note that their work “fails to incorporate the community members’ perceptions of the problem [20]. It is noteworthy that, in April 2013, Six Nations had been under a band-ordered boil water advisory for more than a decade, 86% of drinking water wells were contaminated and 300 homes had no access to treated water [24]. In contrast, residents of the Saskatchewan First Nations community of Muskoday are supplied by water piped in from an off-reserve community supply. As Neegan Burnside Ltd. reported, such Municipal Type Agreement systems are generally classified within the low risk category [8]. Thus, past experience and trust in water supply and supplier may influence risk perceptions related to drinking water. There is empirical evidence in the literature of a significant correlation between prior negative experiences and the acceptability of drinking water quality and risk [17,25]. Enhanced trust and familiarity with the water supply system may be factors supporting the similarities, and in some cases, decreased concern with tap water, which is what we observed between the Muskoday and cross-Canada survey respondents.

Finally, lost control over water resources and traditional lands as a result of land use decisions could also be a factor contributing to differences in observed perceptions of drinking water quality [16,26]. Perceived control is also central to perception of drinking water quality [16]. This often relates to concerns about the adequacy of consultation by government and industry with First Nations on projects that directly affect drinking water in their traditional territories. Culture may play a role. Culture shapes world views, experience, understanding and significance of water. Canter *et al*. [27] suggest that culture influences water perceptions by influencing trust in institutions and ways in which risks are individualized or extrapolated in community. Cultural factors also shape the interpretation of the environment [28,29].

In addition to our interest in comparing First Nations and non-First Nations perceptions and choices, we investigated whether two observable socio-demographic factors (age and gender) played a role. While there were age and gender differences for some of the outcomes studied, there were no differences in the apparent effect of age or gender observed across communities. Recent literature suggests that younger people are more likely to think bottled water was safer than tap water and to spend more on bottled water [17], although in a 2001 survey of two cities in Québec [13], mixed results were found. In our study, younger respondents aged 18–24 were more likely to believe someone had become sick from, and bottled water was safer than, tap water compared to the other two age categories. Younger respondents also reported high monthly bottled water expenditures and greater bottled water use as compared to respondents between ages 25–54 and ≥55. Dissatisfaction in water supply service and the higher risk perception associated with tap water might have contributed to the findings observed in our study among the younger respondents. This could be the result of the widespread broadcasting of the contamination events in Ontario and Saskatchewan during the last decade. Despite this, the youngest age groups were less likely to drink bottled water 100% of the time. Differences in income levels could have potentially led to this result: namely, given their expressed concerns about tap water, young people are more likely than older persons to have high monthly bottled water expenditures; however, their incomes might be insufficient for them to be 100% bottled water drinkers.

Gender effects were similar across all communities and consistent with previous reports in the literature on the relationship between gender and environmental concerns, particularly as they relate to health issues. In particular, other researchers have found significant differences in both perceptions and choices in which women were more likely to perceive risks and act defensively than men [17,19,30]. This finding was supported by our surveys.

There are several limitations to the current study. The first is whether adequate numbers of survey participants were recruited within communities surveyed and the potential for selection bias across communities based on differences in how the survey was distributed. The recruitment email for the non-First Nations study did not indicate details on the subject of the survey, minimizing the potential for selection bias in enrollment. The response rate for the non-First Nations study is 23.5%, which is typical for this type of study [11], and respondents matched Canadian 2006 Census data for age, gender, and income [31]. While there might be more concern for selection bias in the First Nations surveys since community partners solicited participation, the response rate for the Saskatchewan survey was 41%. This is above what is reported in many community-based, observational studies. It was not possible to calculate the response rate for the Ontario surveys due to the method of administration. This is a weakness of the paper. There is very limited information available at the aggregate level pertaining to First Nations populations. Statistics Canada notes that these data are considered less reliable than Census data due to smaller First Nations populations and under coverage that results from populations that are transient [32]. A comparison of these data with the socio-demographic information on respondents in our samples shows that our samples have greater female representation and a greater proportion of the two older age categories.

A second weakness with our study is the potential for unmeasured factors to influence participants’ responses. Such factors may include: income, education level, and marital status. Other studies relating to water and risk perceptions suggest that higher incomes are associated with greater expenses on bottled water while higher education is associated with lower expenditures [17]. Some First Nations partners involved in the research project suggested that the collection of socio-demographic information relating to education level and income was sensitive and might be perceived as conferring judgment on the community. Therefore, this information was not collected. It must be recognized, however, that First Nations communities across Canada are unique.

Finally, the results of the surveys reflect the opinions of the participating communities and we cannot provide general statements about perceptions among Canadian First Nations, particularly recognizing the relatively small number of communities considered as part of this initiative. Nonetheless, the results illustrate that there are a range of views and management circumstances in Canada today.

## 4. Conclusions

This study compared responses to five identical survey questions, developed independently and distributed to diverse groups of individuals living in Canada to gain an understanding of their beliefs of health risks from tap water and corresponding use of and spending on bottled water. It is the first to report on and undertake comparisons of perceptions on drinking water from the perspectives of individuals living in First Nations and non-First Nations communities. Although only four First Nations communities within Canada are represented here, this study does demonstrate for the first time that differences in perceptions of drinking water exist among those surveyed and that these differences could potentially be related to place of residence, experience with, and type of, water supply management, trust and regulation. For example, the Muskoday First Nations community in Saskatchewan receives pre-treated drinking water from a local surface water source while Ontario First Nations communities obtain their water supply either from groundwater or from a very heavily used river that is then treated on-site. The latter have been subject to many boil water orders and these experiences appear to have had a great influence upon perceptions and decisions. This study also demonstrates the need to gain better understanding of the factors that influence perceptions of drinking water quality to better inform and make improvements in water management, supply and monitoring.
